# Perfluorocarbons for the treatment of decompression illness: how to bridge the gap between theory and practice

**DOI:** 10.1007/s00421-019-04252-0

**Published:** 2019-11-04

**Authors:** Dirk Mayer, Katja Bettina Ferenz

**Affiliations:** 1 Department of Gastroenterology, REGIOMED Klinikum Coburg, 96450 Coburg, Germany; 2 Institute of Physiology, CENIDE, University of Duisburg-Essen, University Hospital Essen, Hufelandstr. 55, 45122 Essen, Germany

**Keywords:** Perfluorocarbon, Decompression illness, Non-recompressive therapy, Emulsified perfluorocarbons, Nanocapsules

## Abstract

Decompression illness (DCI) is a complex clinical syndrome caused by supersaturation of respiratory gases in blood and tissues after abrupt reduction in ambient pressure. The resulting formation of gas bubbles combined with pulmonary barotrauma leads to venous and arterial gas embolism. Severity of DCI depends on the degree of direct tissue damage caused by growing bubbles or indirect cell injury by impaired oxygen transport, coagulopathy, endothelial dysfunction, and subsequent inflammatory processes. The standard therapy of DCI requires expensive and not ubiquitously accessible hyperbaric chambers, so there is an ongoing search for alternatives. In theory, perfluorocarbons (PFC) are ideal non-recompressive therapeutics, characterized by high solubility of gases. A dual mechanism allows capturing of excess nitrogen and delivery of additional oxygen. Since the 1980s, numerous animal studies have proven significant benefits concerning survival and reduction in DCI symptoms by intravenous application of emulsion-based PFC preparations. However, limited shelf-life, extended organ retention and severe side effects have prevented approval for human usage by regulatory authorities. These negative characteristics are mainly due to emulsifiers, which provide compatibility of PFC to the aqueous medium blood. The encapsulation of PFC with amphiphilic biopolymers, such as albumin, offers a new option to achieve the required biocompatibility avoiding toxic emulsifiers. Recent studies with PFC nanocapsules, which can also be used as artificial oxygen carriers, show promising results. This review summarizes the current state of research concerning DCI pathology and the therapeutic use of PFC including the new generation of non-emulsified formulations based on nanocapsules.

## Introduction

Sudden change of barometric pressure during underwater ascent or aerospace-related events may lead to a complex of symptoms, clinically diagnosed as decompression illness (DCI), also referred to as divers’ disease, caisson disease or “the bends”. The main mechanism of DCI is the formation of gas bubbles, first described by Robert Boyle in the seventeenth century: “*The little bubbles*…*by choking up some passages, vitiating the figure of others, disturb or hinder the due circulation of blood*” (Acott 1999). In 1841, the clinical syndrome was observed in coal miners who worked in hyperbaric atmosphere (Triger 1845) and also to a greater extent with the use of caissons, watertight pressurized capsules, which allowed underwater construction of bridge piers. During the building of the Eads Bridge (St. Louis) and the Brooklyn Bridge (New York City), 12 workers died and 140 were severely affected (Butler 2004). The frequency of DCI cases significantly increased after the invention of the first successful self-contained underwater breathing apparatus (scuba) by Cousteau and Gagnan followed by increasing use as sports equipment in recreational diving since the 1960/1970s until today (Brubakk and Neuman 2003). Comparing the time periods 1966–1980 and 1999–2013 in Denmark, Juhl et al. found a ten-fold increase in DCI-cases with an average annual case frequency of 14 (Juhl et al. 2016). Thirty-one cases per year were confirmed in New Zealand in 1996–2012 and 274 per year in Australia in 1995–2007 (Haas et al. 2014; Lippmann et al. 2016). A database of 39.099 dives made by 2,629 European divers over 5 years included 320 cases of DCI (0.81%) (Cialoni et al. 2017). Vann et al. estimated that the rate of DCI occurrence per dive varies according to the examined population from 0.015% for scientific divers, 0.010–0.019% for recreational divers, 0.030% for US Navy divers to 0.095% for commercial divers (Vann et al. 2011).

Besides diving, another scenario could be the escape from a distressed submarine. Flooding in this situation increases the ambient pressure and decreases the maximum depth from which leaving the submarine is safe (Jurd et al. 2014). A delay in rescuing the crew leads to saturation with inert gas, thus increasing the probability of DCI (Dainer et al. 2007). With humans venturing into higher layers of the atmosphere and extravehicular activity in space, the syndrome of altitude-related DCI has become progressively more important. The first case was documented in 1862, when Bert described his own neurological symptoms after a hot air balloon ride up to 8838 m (28,000 ft) (Boycott et al. 1908; Bert 1878). Symptoms typically occur while being reexposed to low atmospheric pressure. Compared with diving-related DCI, arterial gas embolism and spinal cord injury are less frequent (Sherman and Sladky 2018).

Originally, perfluorocarbons (PFCs) were developed as inert chemicals to store plutonium and uranium during creation of nuclear weapons in the Second World War (Manhattan project). These synthetically produced perfluorinated carbon compounds are characterized by high energetic bonds and carbon chains completely substituted with halogens. Apart from their very low reactivity, it was soon noticed that high amounts of gases can be dissolved in liquid PFC and Clark captured public’s interest by his spectacular experiment of “liquid breathing” (Clark and Gollan 1966). Cavities between the molecules apparently explain the high solubility of respiratory gases like oxygen, carbon dioxide or nitrogen, in which solubility of nitrogen always exceeds oxygen solubility (Wesseler et al. 1977). Uptake and release of oxygen are twice as fast as that of erythrocytes and directly proportional to oxygen partial pressure, following Henry’s law (Faithfull 1992). Oxygen uptake can even exceed that of haemoglobin if exposed to high oxygen partial pressure. Over 90% of the dissolved oxygen is released into the tissue, three times more compared to the oxygen extraction rate of an erythrocyte (Keipert et al. 1996). This is the reason for the primary use of PFCs as "artificial blood" in different formulations and technologies (Dinkelmann and Northoff 2002; Stephan et al. 2014; Bauer et al. 2010; Spahn 1999; Ferenz 2019a). Furthermore, they are already used for liquid ventilation in newborns (Fuhrman et al. 1991) and for vitreoretinal surgery (Mertens et al. 2000). Improvement in oxygenation also plays an important role in the therapeutic potential of PFC in DCI. Although the reduction in morbidity and mortality in DCI is due to the ability of PFC to dissolve nitrogen from tissues and transport it to the lungs (Spiess 2009), it has been demonstrated that PFC application combined with oxygen breathing significantly increases the clearance of nitrogen-bubbles compared to air and oxygen breathing alone (Riess 2001). This points to the unique and dual nature of PFC which is N_2_ transport from and O_2_ transport to the tissues. Thus, from a theoretical point of view, PFC fulfil many criteria for the ideal non-recompressive therapy in DCI, but this promise was never kept in clinical practice. Although for many years, intravenously administered PFC emulsions have been demonstrated to be effective in rodents and swine models of DCI (Dromsky et al. 2004; Randsoe and Hyldegaard 2014; Dainer et al. 2007; Lynch et al. 1989; Lutz and Herrmann 1984; Spiess et al. 1988), until today, no PFC preparation is accepted for human use by western federal authorities, e.g. the US Food and Drug Administration (Ortiz et al. 2014).

The aim of this review is to summarize the current research findings concerning the use of different PFC preparations in prevention and therapy of DCI and to describe their potential for future developments.

## Methods

Medline was searched up to October 24th, 2019. With the search terms ("fluorocsarbons"[MeSH Terms] OR "fluorocarbons"[All Fields] OR "perfluorocarbon"[All Fields]) AND ("decompression"[MeSH Terms] OR "decompression"[All Fields]), 48 matches were found.

We considered only publications, which offered fundamental new findings or have been regularly quoted in other publications. Studies that confirmed known facts or showed only little consistency were ignored. Systematic reviews and fundamental studies on the subject were added even if not listed under these search terms, if they offered technical or scientific information.

## Main text

### Pathology of DCI and conventional treatment options

**Fig. 1:**
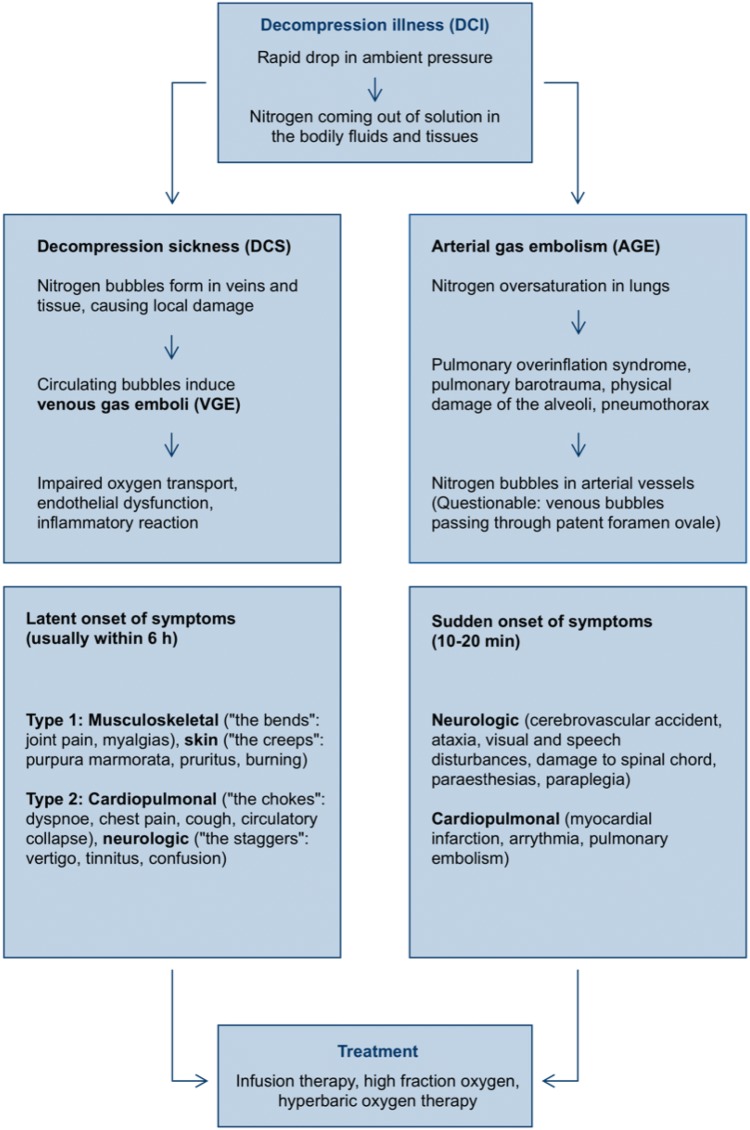
Decompression illness: overview of clinical forms, pathophysiology, symptoms and treatment (modified from (Sykes and Clark 2013)). This figure shows the main pathophysiological mechanisms of decompression illness (DCI) and its main manifestations as decompression sickness (DCS) and arterial gas embolism (AGE). The typical symptoms and degrees of severity are listed. The treatment of both forms of decompression illness is mentioned in short

An abrupt decrease in ambient pressure, for example, in the case of a diver surfacing too quickly or loss of cabin pressure in an airplane, leads to super saturation of tissues with inert gas and formation of nitrogen bubbles (Tikuisis 2003; Eckenhoff et al. 1990). The evolution of these bubbles in vivo is not completely understood. It is suggested that they develop from hydrophobic spots: either micronuclei, very small pre-existing gas bubbles which are adherent to the endothelium, or phospholipidic spots at the luminal surface of the vessels, according to a concept of Arieli (Evans and Walder 1969; Vann et al. 1980; Blatteau et al. 2006; Arieli 2017). Thom et al. demonstrated that intra-microparticle nitrogen functions as a nascent gas nucleation site and can be responsible for initiating post decompression inflammatory injuries (Thom et al. 2012). De novo bubble formation is unlikely to occur, because a pressure gradient (*P*_tissue_N_2_/*P*_ambient pressure_) of several atmospheres would be necessary for this process (Boycott et al. 1908). Within the bloodstream, gas bubbles are coated with proteins interacting with the surroundings (Eckmann and Diamond 2004). The resulting venous gas emboli (VGE) and their subsequent effects on blood vessels, including inflammation, clotting and complement activation (Brubakk and Neuman 2003) seem to be the central mechanisms of decompression sickness (DCS) in the narrower sense, although this pathophysiological theory is not unchallenged (Madden and Laden 2009). Strictly speaking, DCI is a comprehensive term for two different manifestations (see Fig. 1): Apart from VGE, pulmonary barotrauma can cause arterial gas embolism (AGE), associated with gas bubbles in the arterial system resulting in vascular obstruction, endothelial lesions, ischemia and secondary inflammation. Even in the presence of antioxidants, reactive oxygen species and bubbles might lead to embolic or biochemical stress (Wang et al. 2015). It has also been suggested that in the case of patent *foramen ovale* or other right to left shunts, venous bubbles can cause AGE (Bennett et al. 2010). However, a recent study found no apparent correlation between patent *foramen ovale*, the number of detectable brain white matter lesions on magnetic resonance imaging (“Unidentified Bright Objects”) and the results of neuro-psychometric tests (Balestra and Germonpré 2016). Figure 1 provides an overview of the main pathophysiological mechanisms of DCI and its manifestations as DCS and AGE.

DCI shows a broad variety from light symptoms [“the bends”: musculoskeletal pain (type I)] to even life-threatening consequences, lung symptoms (“the chokes”), cardiopulmonary failure and severe damage of the central nervous system, typically affecting the white matter of the spinal cord (type II) (Vann et al. 2011; Hallenbeck 1976). The severity of DCI depends on the degree of direct tissue damage caused by growing bubbles or indirect cell injury by impaired oxygen transport or inflammation. Some studies documented effects of these decompression-induced bubbles on the vascular endothelium (Nossum et al. 1999) or the cerebral circulation, and showed a correlation of overall survival to the amount of emerging nitrogen bubbles. This impact seems to be vessel dependent since more recently Mazur et al. did not find endothelium dysfunction in aortas from rats suffering DCI (Mazur et al. 2016). Since nitrogen bubbles can be detected and observed using ultrasound (Doppler ultrasound monitoring and visual two-dimensional ultrasound imaging), it is assumed that decompression-induced vascular bubbles can be used as a measure of decompression stress and hence decompression safety (Mollerlokken et al. 2012; Eftedal et al. 2007). The link between detection of VGE by ultrasound and DCI was demonstrated by Ljubkovic et al. (2011). However, the extent of VGE is not strictly correlated with the severity of symptoms (Brubakk and Neuman 2003) and endothelial dysfunction (Germonpré and Balestra 2017). On the other hand, “silent”, asymptomatic VGE are more frequent than expected; they can occur in up to 50% of divers after saturation in an immersion depth of only 3.5 m seawater (Eckenhoff et al. 1990). In this respect, significant inter-personal variability has to be considered, too (Papadopoulou et al. 2018). Furthermore, bubble formation plays an important role especially in fatty tissue, but these bubbles are much more difficult to detect and to be measured quantitatively (Randsoe 2016). The effects of non-recompressive therapies on these extravascular bubbles are still unknown.

Conventional treatment of DCI is based on hyperbaric oxygen-breathing combined with recompression followed by successive controlled decompression in a hyperbaric chamber (Bennett et al. 2010). According to the United States Navy Treatment Table 6, the following protocol is most frequently used for serious cases: Maximal pressure of 284 kPa combined with a 100% oxygen breathing schedule lasting 4 h and 45 min, followed by stepwise decompression (Network 2001). The aim of this therapy is to reduce the size and number of gas bubbles by enhancing nitrogen-elimination and to improve the tissue oxygenation (Dart and Butler 1998). This procedure leads to symptomatic relief in 50 to 98% of the patients (Thalmann 1996). The required equipment is expensive, needs well-trained personnel and may not be freely available, e.g. under difficult conditions or in remote locations. Therefore, there is a need for alternative strategies (Spiess 2010). Especially in situations, such as the mentioned distressed submarine or an airplane accident with many simultaneously injured persons, it is obvious that the practical feasibility of recompression therapy is limited. The principles and issues of pre-hospital management of DCI are summarized in a current consensus guideline (Mitchell et al. 2018).

### PFCs in therapeutic use

PFCs are characterized by their high carbon–fluorine bond energy (480 kJ mol^−1^). They are chemically and metabolically inert and produce no toxic degradation products (Riess 2001). Their most outstanding property is an extremely high gas-dissolving capacity. The solubility of respiratory gases is related to the molecular volume of the dissolving gas and decreases in the order CO_2_>> N_2_> O_2_ (Wesseler et al. 1977). In contrast to the active binding of oxygen to heme, the solubility of respiratory gases in liquid PFC is directly proportional to their partial pressure according to Henry’s law. The combination of their ability to enhance oxygen delivery with the facilitation of nitrogen bubble elimination makes PFCs ideal candidates for DCI prevention and treatment (Zhu et al. 2007). The transport capacity of PFC for nitrogen reaches nearly 50 volume percent under normobaric conditions (Spiess et al. 1988; Randsoe 2016). Size and quantity of nitrogen bubbles were shown to be reduced in a cardiopulmonary bypass model (Yoshitani et al. 2006). Furthermore, PFCs function like a surfactant that reduces the adhesion of bubbles to the endothelium and thereby attenuating thrombin production (Eckmann and Diamond 2004; Suzuki et al. 2004). However, PFC preparations cannot be easily administered parenterally. Suitability depends on parameters like retention time in the vascular system, organ retention time and emulsifiability (see “Peculiarities of PFC-based preparations for intravascular use”). This is why PFCs such as perfluorodecalin (C_10_F_18_), perfluoroctylbromide (CF_3_(CF_2_)_7_Br), and perfluorotertbutylcyclohexan (C_10_F_20_) have been mostly used for trials in DCI (see “(Pre-) clinical studies with PFC-based preparations to treat DCS”).

### Peculiarities of PFC-based preparations for intravascular use

Most preparations of PFC are based on emulsion technology, because intravenous injection of high amounts of unprocessed PFC leads to death by spontaneous foaming in the lung (Lanaro et al. 2014). This is mainly because PFCs are neither hydro- nor lipophile and, therefore, immiscible with aqueous fluids like blood. In contrast, very low doses of PFC molecules can be present in the blood without doing any harm: After phagocytosis of the emulsion droplets and association with lipoproteins, small amounts of PFC molecules are transported to the lung, where they can be exhaled if they are characterized by high vapour pressure such as perfluorodecalin (Clark and Gollan 1966; Riess 2001; Lowe 2003). A PFC with exceptional characteristics is dodecafluoropentane (DDFPe). Its boiling point of 29 °C leads to volatilization at biological temperatures. The half-life of DDFPe in systemic circulation is extremely short and it is nearly completely exhaled by the lungs (Johnson et al. 2009).

Although very commonly used, the formulation of a homogenous, sterile emulsion, which is stable at room temperature and characterized by a droplet size of 0.1 to 0.2 µm, is a technical challenge. Under these conditions Ostwald ripening, caused by molecular diffusion, leads to enlargement of the droplets. This process can be counteracted by either adding a small amount of PFC with a higher molecular weight (unfortunately associated with longer organ retention time) or emulsifiers to reduce surface tension (Riess 2005). Highly effective synthetically produced emulsifiers can lead to severe side effects (see below and (Ferenz 2019a; Kuznetsova 2003)). PFC emulsions of the last generation are based on the combination of different emulsifiers such as more tolerable but less stabilizing phospholipids (e.g. egg yolk) with PFCs like perfluorotributylamine (N(CF_2_CF_2_CF_2_CF_3_)_3_) or perfluoromethylcyclohexylpiperidin (C_12_F_22_N) (Ferenz 2019a; Kuznetsova 2003). High molecular weight PFCs, as the latter, are characterized by long persistence in organs resulting in negative decisions from regulatory authorities.

**Fig. 2:**
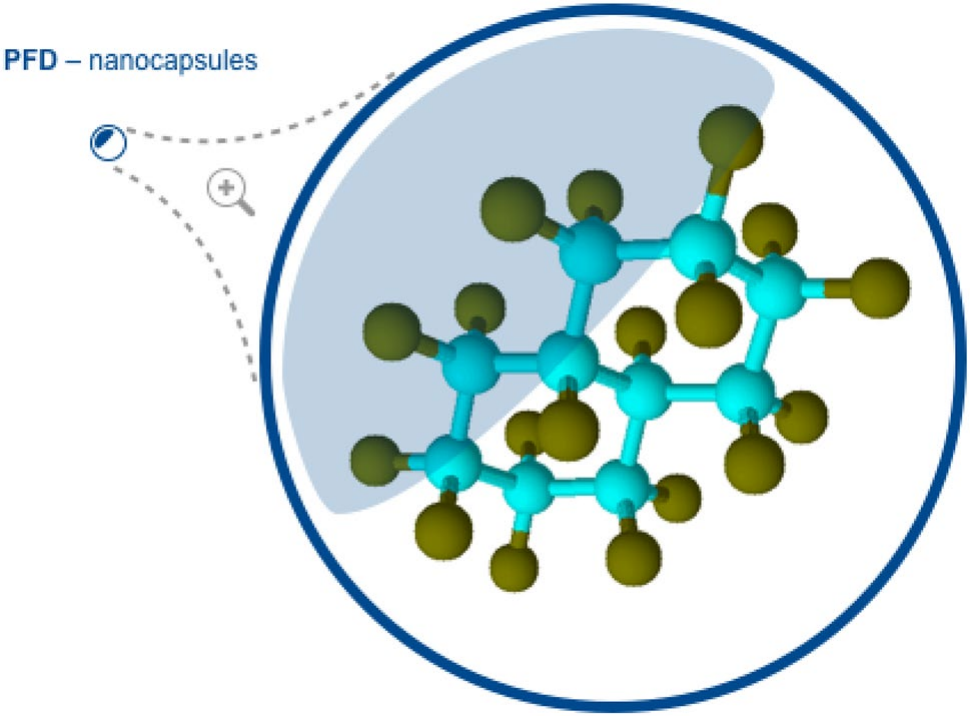
Illustration of an albumin-derived perfluorocarbon-based artificial oxygen carrier. Schematic representation of the albumin shell containing the three-dimensional structure of the perfluorocarbon perfluorodecalin (PFD)

Experience in the last decade shows the need for further development of non-toxic PFC-formulations which can be stored easily and administered directly into the blood stream without relevant side effects. An alternative option to guarantee a stable formulation without Ostwald ripening and the disadvantage of prolonged excretion time is the encapsulation of PFC with polymers, for example poly(lactide-co-glycolide) (Ferenz et al. 2013, 2014) or poly(*n*-butyl-cyanoacrylate) (Stephan et al. 2014; Laudien et al. 2015). Potential areas of application for nanocapsules with a polymer-based shell are controlled drug delivery (Singh and Nalwa 2011; Byagari et al. 2014; Huang et al. 2013; Jiang et al. 2013) and artificial oxygen carriers (Stephan et al. 2014; Laudien et al. 2015; Xiong et al. 2013). The thin capsule wall provides compatibility with the aqueous medium blood and allows easy release or absorption of respiratory gases (Stephan et al. 2014). Our recent toxicity studies showed insufficient biocompatibility of poly(*n*-butyl-cyanoacrylate) (Laudien et al. 2015) (see “Reasons for failure of PFC-based preparations in clinical trials”). A new solution is the combination of the amphiphilic biopolymer albumin, characterized by lacking toxicity and antigenicity (Patil 2003), with PFD, the PFC particularly suitable for medical purposes. Those albumin-derived artificial oxygen carriers (A-AOCs) revealed a higher oxygen transport capacity than Perftoran™ and tolerable side effects (Wrobeln et al. 2017b) (see “Albumin-derived artificial oxygen carriers: a new option”). Figure 2 shows a schematic illustration of an albumin-derived perfluorocarbon-based artificial oxygen carrier.

### (Pre-) clinical studies with PFC-based preparations to treat DCS

All PFCs used for experimental treatment of DCS are summarized in Table 1.

**Table 1:** Different PFC preparations (modified from Castro and Briceno 2010)

Product name	Company	PFC-composition	Emulsifier	Storage	Reasons for missing approval	(pre-)clinical studies
Fluosol-DA™ (FG)	Fluosol-DA, Green Cross Corp., Osaka, Japan; Alpha Therapeutic, Los Angeles, CA, USA	14% perfluorodecalin, 6% perfluorotripropylamine	Pluronic F-68, egg yolk-phospholipids, potassium oleate	frozen	Insufficient stability, long organ retention time of 65 days	(Ingram et al. 1993; Lutz and Herrmann 1984; Mattrey et al. 1989; Riess 2001)*, (Lane 1995; Vercellotti et al. 1982; Young et al. 1990)**
Oxypherol™/Fluosol-43™/FC 43™ (FG)	Fluosol-DA, Green Cross Corp., Osaka, Japan; Alpha Therapeutic, Los Angeles, CA, USA	20% perfluorotributylamine	Pluronic F-68	not known	Extremely long organ retention time, half-life in the rat about 2.5 years	(Bito et al. 2000; Lynch et al. 1989; Ochikubo et al. 1999; Spiess et al. 1988; Spiess et al. 1986)*
Perftoran™/Vidaphor™ (FG)	FluorO2 Therapeutics, Boca Raton, Florida; Ftorosan, OJCS SPF Perftoran Russian, Moscow, Russia	1% perfluorodecalin, 3% perfluoromethylcyclohexyl-piperidin	Proxanol 268, egg yolk-phospholipids	3 years frozen (−4 to −18 °C)	Long organ retention time of 90 days (approval only in Russia, Ukraine, Kazakhstan Kyrgyzstan, Mexico)	(Eckmann and Lomivorotov 2003; Leskova et al. 2003)* (Maevsky et al. 2005)**
Oxygent™ (SG)	AF0144, Alliance Pharmaceutical Corporation, San Diego, CA, USA; Double Crane Pharm. Co., Beijing, China	58% perfluoroctylbromid, 2% perfluorodecylbromide	egg yolk-phospholipids	1–2 years, 5–10 °C	Severe side effects such as ileus and increased frequency of strokes	(Dainer et al. 2007; Dromsky, Spiess and Fahlman 2004; Mahon et al. 2010; Zhu et al. 2007)* (Keipert 2006; Leese et al. 2000; Spahn et al. 2002)**
Oxycyte™ (SG)	Tenax Therapeutics, Inc., Morrisville, NC (Synthetic Blood Int. Inc.)	60% perfluorotertbutylcyclohexan	egg yolk-phospholipids	not known	Sponsor withdrew support	(Smith et al. 2012; Yoshitani et al. 2006)* (Winslow 2006)**
Oxyfluor™ (SG)	HemaGen Inc, St. Louis, MO	78% perfluorodichlorooctane	egg yolk-phospholipids, safflower oil	1 year, room temp	Phase III clinical trials suspended	(Briceño et al. 1999; Cochran et al. 1997)* (Winslow 2006)**

Overview of emulsified perfluorocarbon (PFC) preparations officially produced at least for a limited period. Specified are the company, the composition, the used emulsifier, conditions of storage, reasons for missed or withdrawn approval and preclinical or clinical studies including summaries of clinical studies

FG first-generation PFC, SG second-generation PFC

*Preclinical studies

**Clinical studies or summary

First studies from the 1980s with rodents revealed the life-prolonging effect of PFC in simulated air dives (Lynch et al. 1989; Lutz and Herrmann 1984). Rats treated with Fluosol-43™ in combination with 100% oxygen after diving showed a significant longer survival without neurologic deficits and an absolute higher survival rate compared with 6% hydroxyethyl starch treatment (control group) (Spiess et al. 1988). Experiments with rabbits also demonstrated a significant survival benefit in the PFC pre-treated group after triggering venous gas embolism, indicating gas absorptive properties of this substance (Spiess et al. 1986). It took until the 1990s before experiments using Oxygent™ (at that time Alliance Pharmaceuticals Inc, San Diego, CA, USA, now Beijing-DoubleCrane Pharmaceuticals, China) in a swine-model delivered new findings confirming a dramatic reduction of DCI lethality. Animals received Oxygent™ intravenously in combination with oxygen and corticosteroids immediately after diving. PFC decreased and delayed the onset of cardiopulmonary DCI and prevented neurological symptoms (Dromsky et al. 2004). A preventive effect of PFC in a dog model of AGE was described by Arnold et al. who found less cerebral strokes and improved cerebral blood flow (Arnold et al. 1993). Further studies in animal models with VGE and AGE consistently confirmed the resorptive capabilities of PFC (Cochran et al. 1997; Lundgren et al. 2005; Yoshitani et al. 2006). Using a Russian preparation (Perftoran™), Eckmann et al. demonstrated a protective effect of PFC against air bubble damage in cultured endothelial cells (Eckmann and Lomivorotov 2003). In a dog model, an infusion of a perfluorodecalin–glycerol emulsion was able to enhance the off-gassing of xenon from muscle tissue (Novotny et al. 1993). Further studies in rabbits led to the conclusion that PFC promotes the pulmonary elimination of nitrogen and, thus, increases the elimination of the bubbles (Zhu et al. 2007).

Another interesting effect was found in sheep experiments, i.e. perfluorotertbutylcyclohexan application increased oxygen delivery to and -utilization in ischemic tissues, which can be considered as the second main pillar in DCI-therapy (Smith et al. 2012). For successful therapy of DCI with PFCs, the correct time frame of PFC application is probably of great importance. Preventive administration of PFC at depth before decompression in a swine model did not lead to better results in a study of Dainer et al.; best efficacy was achieved by the combination of PFC after diving with breathing 100% oxygen (Dainer et al. 2007). In a study from 2010, Mahon et al. confirmed these results. They tested the efficacy of Oxygent™ not immediately post-dive but with a time lag after onset of DCS in combination with oxygen and demonstrated that even delayed application of PFC is effective in decreasing mortality in swine (Mahon et al. 2010).

But not every PFC preparation appears to be useful. In a recent study by Sheppard et al. dodecafluoropentane (DDFPe) was associated with a high mortality and showed no beneficial effects in a rat model with DCS (Sheppard et al. 2015). Randsoe et al. demonstrated that under normobaric conditions, the combination of oxygen breathing and PFC leads to accelerated reduction of bubbles and that both therapies complement each other (Randsoe and Hyldegaard 2009). The initial bubble growth caused by increased oxygen tension is only transient and compensated by the passive transport capacity of PFC. The additional use of a hyperbaric chamber to further boost the positive effect of PFCs was of no further benefit. In contrast, the combination of breathing highly concentrated oxygen with PFC as a substance with high oxygen capacity appears to be dangerous at depth. Mahon et al. showed in a swine model at 507 kPa a significant increase of seizures versus the control group with saline infusion (Mahon et al. 2006). Further studies are necessary to assess if a combined PFC-oxygen therapy at lower pressures can avoid toxic oxygen effects with higher efficacy than PFC alone (see “Reasons for failure of PFC-based preparations in clinical trials” risk of seizures).

### Reasons for failure of PFC-based preparations in clinical trials

Although physico-chemical properties of PFC-based preparations appear to make them ideal candidates for DCI therapy and many preclinical studies apparently confirm this concept, until today, no broad success in clinical practice has been achieved. Table 1 provides an overview of at least temporarily commercially available PFC-preparations and their main reasons for rejection by official authorities.

One fundamental and recurrent problem of PFC emulsions is their *long organ retention time*, which can be associated with an incalculable long-term effect. Further development of Oxypherol™, a stable emulsion based on a combination of perfluorotributylamin with Pluronic F-68, was discontinued for this reason (Riess and Krafft 2006). Perftoran™ is the only PFC preparation with limited approval by official authorities in Russia, Ukraine, Kazakhstan, Kyrgyzstan and Mexico (Castro and Briceno 2010). The main indications are acute blood loss, improvement of oxygenation of specific tissues for example in coronary heart disease, ischemic disorders of extremities and brain, acute or chronic anaemia and wound healing (Maevsky et al. 2005, 2006). Perftoran™ is generally well tolerated; rarely occurring side effects are dizziness, kidney pain, hypotension, hyperaemia, lung symptoms and temporary itching (Maevsky et al. 2005). Its long organ retention time (90 days) caused by the additive of perfluoromethylcyclohexylpiperidine (Castro and Briceno 2010) and non-conform production process resulted in refused approval of Perftoran™ in Europe and USA. Currently, there are efforts to produce Perftoran™ under good clinical practice conditions and thus to gain U.S. Food and Drug Administration approval under the brand name Vidaphor™ (Latson 2017).

Further issues concern *lack of stability and difficult handling* of the substance. At the beginning of the 1990s, Fluosol-DA™ obtained approval for improving oxygenation during coronary angioplasty in USA, Europe and Japan (Lowe 2003, 2006; Kocian and Spahn 2008; Castro and Briceno 2010). Only a few years later, it was removed from the market not only because of its very long organ half-life but also because of its insufficient stability. Storage at − 20 °C associated with a long defrosting period limited its usability (Riess 2001) and production of Fluosol-DA™ was definitively stopped in 1994 (Lowe 2006). Enhanced PFC products of the second generation are characterized by storage without freezing, 2–4 times higher PFC contents and natural phospholipids as emulsifiers (Castro and Briceno 2010).

But also, these further developed preparations failed in practice. In some cases, *relevant side effects* were the main reason for the termination of clinical studies. Several phase-II studies with Oxygent™ including patients with cardiac surgery or orthopaedic interventions in combination with haemodilution have shown promising results (Keipert et al. 1996; Castro and Briceno 2010). Two phase-III studies in Europe, USA and Canada reported a reduced number of transfusions but also severe side effects such as post-surgical ileus and an increased frequency of strokes. A post hoc analysis could not confirm this correlation, but the sponsor stopped the study (Keipert 2006). In 2005, another study with Oxygent™ in cardiac surgery patients investigating brain circulation found increased neurological complications such as cerebral emboli. However, the detection technique was based on ultrasound Doppler, which could be a limitation in this setting and responsible for the negative outcome in the Oxygent-treated patients (Hill et al. 2005). Since 2017, Oxygent™ is approved in China for clinical studies in humans, financed by Double Crane Pharm. Co. (Beijing, China) (Ferenz 2019b; Riess 2006).

Sometimes *unspecific non-medical reasons* lead to premature termination of clinical trials. One of the most promising PFC preparations Oxycyte™ was tested in a phase-II-study including patients with severe traumatic brain injury. The study started in 2009 and was terminated by the sponsor in 2014, due to problems with patient recruitment. The sponsor then withdrew his support of the product (Winslow 2006). To our knowledge since 2014, no other study with this PFC emulsion has been performed.

There are concerns about the *risk of seizures* due to toxic oxygen effects if PFC therapy is combined with the use of hyperbaric oxygen, eventually caused by the high oxygen carrying capacity of PFC. In a recently published mixed swine study, Cronin et al. found no increased rate of seizures after receiving PFC and following recompression with hyperbaric oxygen (Cronin et al. 2018).

Several studies in different animal species showed an *increase in pulmonary arterial pressure* (PAP) (Hall et al. 1985; Spiess et al. 2009; Smith et al. 2012). This observation, for example in sheep, is even more relevant, because severe DCI itself can cause high PAP due to nitrogen-bubble-associated mechanical obstruction or indirectly by endothelial effects with vasoconstriction (Josephson 1970; Sheppard et al. 2015). A recent mixed-sex swine study using the emulsified perfluorocarbon Oxycyte™ revealed a prolonged PAP increase in this species (Mahon et al. 2015). There is one report of an adverse pulmonary reaction in human use concerning Fluosol™ (Vercellotti et al. 1982). A possible explanation for this side effect of PFC emulsions could be a *complement activation pseudoallergy* (CARPA) caused by retention of lipid particles >0.35 µm in the lung that consecutively activate the complement system (Szebeni et al. 2000; Sheppard et al. 2015). PFC emulsions like Oxycyte™ with a particle size up to 0.6 µm (median size 0.2–0.25 µm) could trigger CARPA. For A-AOCs, characterized by a mean diameter of 0.4 up to 0.7 µm (depending on the dispersion medium) (Wrobeln et al. 2017b), the occurrence of CARPA is also possible. Thus, it must be considered that nanocapsules-based PFC preparations could also lead to an additional increase of PAP. The potential of PFCs causing PAP via CARPA should be kept in mind, if future studies on PFCs in DCI treatment again reveal increased PAP.

Keipert et al. demonstrated a *febrile reaction* up to 1–1.5 °C (6–8-h duration) in rats after intravenous application of a concentrated emulsion of Oxygent™. Intensity and duration of the fever attacks showed inverse dependency on particle size. The authors concluded that emulsion particles < 0.2 µm are associated with a longer half-life period in blood, less activity of macrophages and thus reduced temperature response (Keipert et al. 1994).

Intravenous infusion of nanocapsules with a polymer-based shell, for example poly(lactide-*co*-glycolide) or poly(*n*-butyl-cyanoacrylate) is in general well tolerated by animals, but side effects can occur, e.g. transient decrease in mean arterial blood pressure, impairment of hepatic microcirculation, organ/tissue damage of liver, spleen and small intestine, elevation of plasma enzyme activities such as lactate dehydrogenase, creatine kinase and aspartate aminotransferase. Accumulation of polymer-based shell nanocapsules in spleen, kidney and small intestine can be assumed; the organ most affected depends on the shell material used (Laudien et al. 2015). The development of more suitable shell materials should combine the favorable gas exchange properties of PFC-nanocapsules with a low risk of potential long-term toxicity.

### Albumin-derived artificial oxygen carriers: a new option

Albumin-derived artificial oxygen carriers (A-AOCs) were designed in search of an artificial oxygen carrier based on nanocapsules technology with properties in terms of biocompatibility and gas exchange comparable to emulsions (Wrobeln et al. 2017b, c). The synthetic procedure entails using ultrasound in the presence of albumin. Amphiphilic albumin as shell material encloses a core of PFD, thus avoiding the requirement of an additional emulsifier. In vitro tests have shown effective oxygen transport capacity of A-AOCs (Wrobeln et al. 2017b), further supported by the proof of functionality in the Langendorff-heart-model (Wrobeln et al. 2017c) and in vivo studies of the rat regarding toxicity and pharmacokinetics (Wrobeln et al. 2017a). Intravenous application of A-AOCs was well tolerated in rats without change in systemic parameters and with stabile vascular perfusion. Parameters of tissue injury showed no relevant deviations and the half-life of A-AOCs (158 min) was sufficient (Wrobeln et al. 2017b). An in vivo proof-of-concept-study then demonstrated survival of rats after progressive exchange of 95% of blood with A-AOCs (Wrobeln et al. 2017a).

**Fig. 3:**
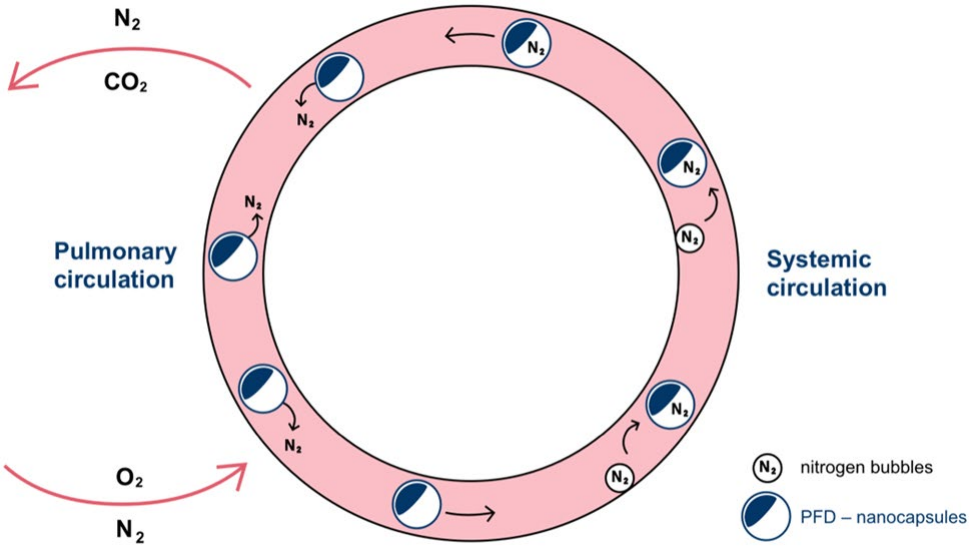
Representation of the principle nitrogen-reducing function of albumin-derived perfluorocarbon-based artificial oxygen carriers in decompression illness (DCI). This figure demonstrates the ability of perfluorocarbon (PFC) containing nanocapsules to capture nitrogen from bubbles adherent to the endothelial wall and transport it to the lungs, where it is exhaled (Sykes and Clark 2013)

In a recent study, the preventive intravenous application of A-AOCs before air diving proved to be well tolerated and effective in reducing the occurrence of DCI. Treated animals showed significantly higher survival rate, longer survival time and less symptoms compared to the group which received serum albumin only. These positive results were confirmed by analysis of histological examinations and of the quickly responding plasma parameters lactate and myoglobin. Interestingly, oil-filled nanocapsules without PFC also showed a non-significant but measurable beneficial DCI-preventing effect. The experiments were performed using an established animal model in cooperation with the French working group of Francois Guerrero, University of Brest (Mayer et al. 2019). The elimination of nitrogen bubbles is not only based on the permeability of the nanocapsules shell material but also the gas exchange capacity of PFC. There is some evidence for a stabilization of nitrogen bubbles by nanoparticles based on the Pickering effect (Du et al. 2003; Lam et al. 2014). It is assumed that nanocapsules cover the surface of small nitrogen bubbles in the nascent state and stabilize them in aqueous dispersion. Hereby, they can prevent further agglomeration and subsequent growth of the bubbles and enable their effective transport in the blood plasma. In this way, the Pickering effect adds to the beneficial capability of the A-AOCs to carry nitrogen in the dissolved state and explains the reduced expression of decompression illness observed also after application of PFC-free oil-filled nanocapsules (Mayer et al. 2019). Figure 3 demonstrates the ability of PFC containing nanocapsules to capture nitrogen from bubbles adherent to the endothelial wall and transport it to the lungs, where it is exhaled.

## Conclusion

Chemical characteristics of PFC offer ideal properties for therapeutic use in DCI and numerous animal studies have shown facilitation of nitrogen bubble elimination in combination with enhanced oxygen delivery. Most of these studies were performed with PFC preparations based on emulsions, which are also used as artificial oxygen carriers in human trials including patients with acute or chronic anaemia. Until today, no PFC emulsion has been accepted for human use by health authorities in Europe and USA. Reasons for the refusal of these formulations pertain to side effects mainly caused by the added emulsifiers, handling difficulties and long organ retention time. Only in some countries outside the western hemisphere, approval of one product (Perftoran™) is granted for use as an artificial oxygen carrier but not for DCI treatment. A new generation of PFC formulations based on nanocapsules could provide the potential to promote non-recompressive drug therapy for DCI. Intravenously administrable A-AOCs combine good tolerability and easy handling with the well-known efficacy of PFD. First in vitro and in vivo tests showed promising results supported by ongoing preclinical trials. PFC-based nanoparticles could offer the chance to bridge the time for a DCI patient until (if any) hyperbaric therapy is available or to act as an additive to mitigate the clinical syndrome. Hopefully, the transfer of this new promising therapeutic option to treat DCI patients will be successful in the near future.

## Abbreviations

AGE: Arterial gas embolism
A-AOCs: Albumin-derived perfluorocarbon-based artificial oxygen carriers
CARPA: Complement activation pseudoallergy
DCI: Decompression illness
DCS: Decompression sickness
DDFPe: Dodecafluoropentane
N_2_: Nitrogen
P: Pressure
PAP: Pulmonary arterial pressure
PFC: Perfluorocarbons
PFD: Perfluorodecalin
VGE: Venous gas emboli
